# An Update on the Role of Androgens and Androgen Receptor in Triple-Negative Breast Cancer

**DOI:** 10.3390/cells15090834

**Published:** 2026-05-02

**Authors:** Belen Crespo Cortes, Felisbina L. Queiroga, Juan Carlos Illera, Sara Caceres Ramos

**Affiliations:** 1 Department Animal Physiology, Veterinary Medicine School, Complutense University of Madrid (UCM), 28040 Madrid, Spain; belencre@ucm.es (B.C.C.); sacacere@ucm.es (S.C.R.); 2 CECAV, Department of Veterinary Sciences, University of Trás-os-Montes and Alto Douro, 5001-801 Vila Real, Portugal

**Keywords:** triple-negative breast cancer, androgen receptor, sex steroid hormones, endocrine therapies, androgens, animal models, cellular processes

## Abstract

Androgen receptor (AR) signaling has emerged as a potential molecular target in triple-negative breast cancer (TNBC), a clinically aggressive and biologically heterogeneous subtype of breast cancer with limited targeted treatment options. Androgens, the main ligands of AR, have been reported to exert antiproliferative and anti-estrogenic effects in normal mammary epithelium; however, the role of AR signaling in TNBC remains controversial and appears to depend strongly on tumor molecular context. In certain experimental settings, elevated androgen levels have been associated with reduced tumor growth, whereas AR activation has also been linked to signaling pathways involved in cell survival, migration, and invasiveness. AR signaling can occur through classical androgen-dependent mechanisms, as well as through ligand-independent activation mediated by protein kinases and intracellular pathways. Increasing interest in AR biology has led to the evaluation of several anti-androgen therapies in AR-positive TNBC, including agents such as enzalutamide, enobosarm, orteronel, bicalutamide, and seviteronel. Although clinical activity has generally been modest, these studies highlight the potential relevance of AR-targeted strategies in selected patient subgroups. This review summarizes current knowledge on androgen and AR signaling in TNBC, integrating molecular mechanisms, preclinical evidence, and clinical studies, and discusses emerging therapeutic strategies aimed at improving patient treatment outcomes.

## 1. Introduction

Triple-negative breast cancer (TNBC) is a clinically aggressive and molecularly heterogeneous subtype of breast cancer accounting for approximately 10–15% of all cases [1,2]. It is characterized by the absence of estrogen receptor α (ERα), progesterone receptor (PR), and the lack of overexpression of human epidermal growth factor receptor 2 (HER2), which precludes the use of conventional endocrine therapies commonly used in hormone receptor-positive breast cancer [2]. Consequently, chemotherapy and surgery currently remain the standard treatment options for patients with TNBC [1].

In an effort to improve therapeutic outcomes, recent research has focused on the identification of novel molecular targets. Among these, the androgen receptor (AR) has emerged as a promising candidate [3]. AR is expressed in a substantial proportion of TNBC tumors, although reported frequencies vary depending on detection methods and molecular subtype [1]. AR-positive TNBC has been associated with distinct molecular characteristics, particularly within the luminal androgen receptor (LAR) subtype, suggesting a unique biological behavior compared with other TNBC subtypes [4].

From a clinical perspective, AR expression in TNBC has been associated with distinct clinic–pathological features, including lower histological grade, reduced proliferative index, and enrichment within the LAR molecular subtype [5]. However, its prognostic significance remains controversial, with studies reporting both improved and worse clinical outcomes depending on patient cohorts and methodological differences in AR assessment [6,7]. Importantly, AR expression has also been explored as a predictive biomarker for response to anti-androgen therapies, although clinical trials have demonstrated modest and heterogeneous results [7]. These findings highlight the need for a more precise characterization of AR-driven tumors and support continued investigation of AR as both a biomarker and therapeutic target in TNBC.

Preclinical studies have supported a role for AR signaling in tumor growth, differentiation, and survival, providing a strong biological rationale for therapeutic AR inhibition and leading to the development of several AR-targeted clinical trials [4,8].

However, the precise role of AR in TNBC progression remains poorly understood [8]. Therefore, elucidating the contribution of androgens and AR signaling in TNBC may facilitate the development of more effective and personalized therapeutic strategies for this breast cancer subtype.

Given the increasing interest in AR signaling as a potential therapeutic target in TNBC, this review aims to provide an integrated overview of current knowledge on androgen biology, AR signaling mechanisms, and emerging AR-targeted therapeutic strategies in this disease. Relevant literature was identified through searches in the PubMed database using the following keywords: “triple-negative breast cancer,” “androgen receptor,” “sex steroid hormones,” “anti-hormone therapies,” and “anti-AR therapies.” In addition, completed and ongoing clinical trials were identified using the ClinicalTrials.gov platform.

## 2. Steroid Hormone Biosynthesis and Androgen Signaling in Breast Tissue

Steroid hormone biosynthesis involves multiple metabolic pathways, with cholesterol serving as the common precursor for all steroid hormones [9]. These pathways are particularly relevant in hormone-dependent malignancies, including breast cancer, where local steroid metabolism can influence receptor-mediated signaling [9]. Under physiological conditions, steroidogenesis predominantly follows the classical Δ5 pathway, leading to the production of androgens and estrogens through sequential enzymatic reactions mediated by cytochrome P450 enzymes, hydroxysteroid dehydrogenases, the aromatase enzyme, and the 5α-reductase family [9,10,11,12,13].

In the Δ5 pathway, cholesterol is converted into pregnenolone and subsequently into dehydroepiandrostenedione (DHEA) and androstenedione (A4), which act as key precursors for testosterone and its more potent metabolite, 5α-dihydrotestosterone (DHT) [10,11,12,13,14,15]. Androgens can also be aromatized into estrogens, highlighting the relation between androgenic and estrogenic signaling [16,17]. This balance between androgen and estrogen synthesis is particularly relevant in breast tissue, where alterations in local steroid metabolism may influence receptor-mediated signaling pathways [12].

A substantial proportion of steroid hormones circulate in sulfated forms, such as DHEA sulfate (DHEA-S) and estrone sulfate (E1SO4), which function as biologically inactive reservoirs that can be locally converted into active hormones by steroid sulfatase (STS) [9,11,18]. This reversible sulfation and desulfation system plays a critical role in regulating tissue-specific steroid availability and activity and has been implicated in hormone-dependent malignancies, including breast cancer [18].

**Figure 1:**
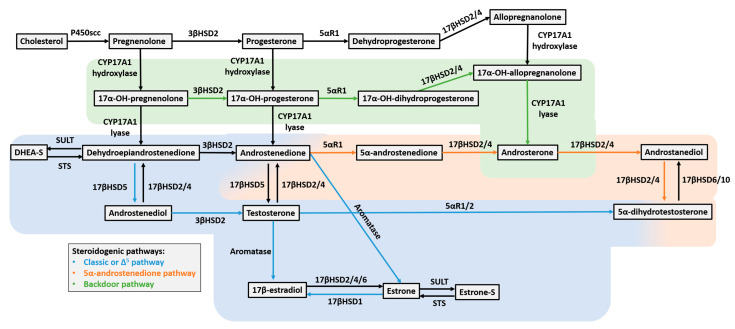
Schematic overview of the steroid hormone synthesis pathways. The classical (Δ5) pathway is highlighted in blue, the 5α-androstenedione pathway in orange, and the backdoor pathway in green. Enzymes catalyzing each reaction are listed alongside the arrows. Figure based on [12].

In addition to the classical pathway, alternative pathways of androgen biosynthesis, such as the backdoor and alternative 5α-androstenedione pathways, have been described and may become relevant under specific physiological or pathological conditions [9,19,20]. These pathways further contribute to the complexity of local androgen production and may partially explain discrepancies between circulating androgen levels and intratumoral androgen activity reported in breast cancer subtypes, including TNBC [12] [Figure 1].

### 2.1. Contribution of Steroidogenic Organs and Physiological Role of Androgens in the Mammary Gland: Relevance for Breast Cancer

In women, androgen biosynthesis arises primarily from the adrenal glands and ovaries, while peripheral tissues, including the mammary gland, possess the enzymatic machinery required for local intracrine steroid metabolism [9,12,21,22,23,24,25,26]. Circulating adrenal-derived precursors, such as DHEA and A4, can be converted into bioactive androgens within breast tissue, thereby modulating local hormone availability independently of systemic endocrine levels [9,12].

Following menopause, ovarian steroidogenesis declines markedly, and adrenal-derived androgen precursors become the predominant source of circulating androgens, further increasing the importance of peripheral steroid conversion within breast tissue [9,27,28]. This intracrine steroidogenic capacity may influence tumor biology even in the absence of classical hormone receptor expression.

Under physiological conditions, androgens exert predominantly antiproliferative and antiestrogenic effects in the normal mammary gland. Through paracrine and autocrine mechanisms, androgen signaling modulates estrogen-driven epithelial proliferation and contributes to mammary gland development and tissue homeostasis [29,30,31].

Although TNBC lacks expression of ERα, PR, and HER2 and has therefore traditionally been considered hormone-independent [27,28], emerging evidence suggests that local steroid metabolism and alternative hormone receptor pathways may still influence tumor behavior [27,30]. The preservation of intracrine androgen production within breast tissue highlights the potential relevance of androgen signaling in the molecular context of TNBC [21].

### 2.2. Androgen Signaling and Its Role in Triple-Negative Breast Cancer

The absence of ERα and PR expression, together with the lack of HER2 overexpression, has traditionally led TNBC to be considered a hormone-independent tumor subtype [32]. However, accumulating evidence has demonstrated that TNBC cells are capable of producing steroid hormones and modulating local steroid metabolism, which may play a crucial role in tumor progression [33]. Moreover, the expression of AR, ERβ, and G protein-coupled estrogen receptor 1 (GPER-1) receptors has reinforced the idea that steroid hormone signaling may influence TNBC biology despite the lack of classical endocrine targets [33,34].

Recent research has focused on the impact of dysregulated steroid hormone synthesis on TNBC development and progression [35,36]. In particular, alterations in androgen and estrogen secretion have been shown to modulate tumor behavior [37]. The role of estrogens in TNBC remains under investigation, although several studies suggest that estrogen signaling pathways may contribute to tumor progression in specific contexts [35,38]. By contrast, the role of androgens has been less thoroughly investigated, although emerging evidence supports a relevant role in TNBC biology [39]. Experimental studies have suggested that increased androgen levels may exert antiproliferative effects in certain TNBC cell models [36]. Similarly, studies using xenograft mouse models of TNBC have demonstrated that increased intratumoral androgen concentrations can inhibit tumor growth [35,38,40]. These findings support the notion that androgens may modulate TNBC progression in a context-dependent manner.

As previously described, androgens can be aromatized into estrogens or reduced to DHT, the main ligand of AR [9,16]. Consequently, elevated androgen levels may either promote estrogen synthesis or exert direct biological effects through AR activation [16]. While substantial efforts have focused on assessing AR expression in TNBC, consideration of local steroid hormone synthesis, secretion, and the balance between androgens and estrogens is equally important. The availability of AR ligands may critically influence receptor activation and downstream signaling, ultimately impacting tumor progression [36].

## 3. The Androgen Receptor in TNBC: Biological Functions and Therapeutic Potential

### 3.1. Androgen Receptor Signaling in Triple-Negative Breast Cancer

**Figure 2:**
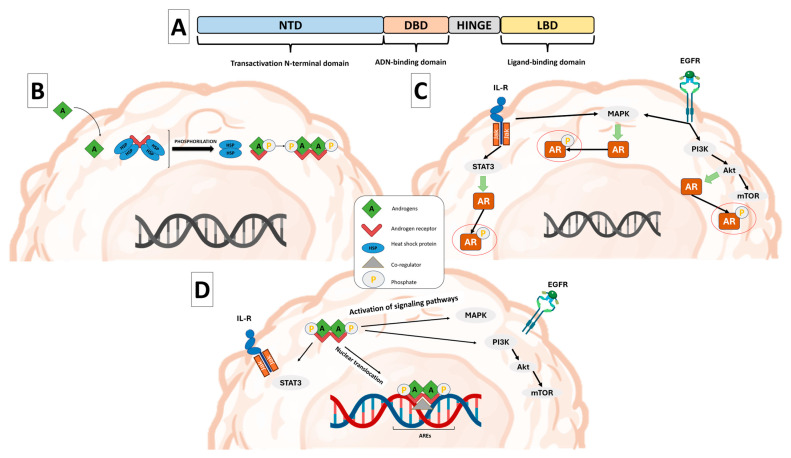
Schematic overview of AR domains (**A**), ligand-dependent activation (**B**), ligand-independent activation (**C**), and AR mechanisms of action (**D**). The legend was added in the central panel. Figure based on [8].

The AR is expressed in a subset of TNBC tumors, with reported frequencies ranging from approximately 10 to 43% depending on detection methods and molecular subtype [8,41,42]. AR belongs to the nuclear steroid hormone receptor superfamily. Structurally, AR comprises an N-terminal domain (NTD) involved in transcriptional regulation; a DNA-binding domain (DBD), responsible for interaction with androgen response elements (AREs); a hinge region; and a ligand-binding domain (LBD) responsible for androgen interaction [43] (Figure 2A).

AR activation can occur through ligand-dependent mechanisms, following binding of androgens such as testosterone or DHT, or through ligand-independent mechanisms mediated by growth factor signaling or kinase-driven pathways [8,41,44,45] (Figure 2B,C). Ligand-independent activation of AR may occur through phosphorylation mediated by kinase signaling pathways such as PI3K/Akt, MAPK, or Src, which can enhance AR transcriptional activity in the absence of androgens. In addition, growth factor signaling has been shown to promote AR nuclear localization and transcriptional function independently of ligand binding. These mechanisms may be particularly relevant in TNBC, where circulating androgen levels are relatively low, allowing tumor cells to sustain AR signaling through alternative activation pathways [46]. Regardless of the activation pathways, AR signaling can activate multiple downstream effector pathways. Activated AR can translocate to the nucleus and regulate gene transcription through AREs or elicit rapid non-genomic responses by modulating intracellular kinase signaling cascades [8,16,42]. These genomic and non-genomic actions are not mutually exclusive and collectively regulate processes such as cell proliferation, survival, and migration [8] (Figure 2D). In this context, AR transcriptional activity is further modulated by interactions with coactivators and coregulators, including steroid receptor coactivators (SRC-1, SRC-2, and SRC-3) and p300/CBP, which facilitate chromatin accessibility and recruitment of the transcriptional machinery [47]. Dysregulation of these factors may alter AR signaling output and contribute to tumor progression and therapeutic resistance, adding an additional layer of complexity to AR-driven pathways that is likely relevant in TNBC [48].

Accumulating evidence indicates that AR signaling plays a context-dependent role in TNBC biology [49]. While AR activation has been associated with increased proliferation, invasion, and metastatic potential in selected experimental models, particularly within the LAR subtype characterized by high AR expression, AR-positive TNBCs have also been linked to more differentiated tumor features and lower proliferative indices in clinical cohorts [5,7,41,42,48,50,51,52,53]. These seemingly contradictory observations suggest that AR function is strongly influenced by tumor molecular context, including TNBC biological heterogeneity, particularly the enrichment of AR signaling within the LAR subtype, as well as differences in ligand availability, co-regulator expression, and crosstalk with signaling pathways such as PI3K/Akt [51]. Together, these factors may explain why AR activation can be associated with either tumor-suppressive or tumor-promoting effects, depending on the molecular context.

Notably, the AR function in breast cancer is highly context-dependent. In estrogen receptor-positive breast cancer, AR activation has been shown to exert tumor-suppressive effects, as demonstrated by Hickey et al. (2021), highlighting that the biological role of AR may differ substantially across breast cancer subtypes [54].

In this context, the biological role of AR appears to differ across TNBC molecular subtypes. In the LAR subtype, characterized by high AR expression and luminal gene signatures, AR signaling is thought to play a more central, potentially oncogenic role. In contrast, in non-LAR TNBC subtypes, AR expression is often lower and its functional contribution less defined, suggesting a more limited or context-dependent biological impact [55,56].

Crosstalk between AR and other signaling pathways further contributes to this complexity. Interactions with estrogen receptor β (ERβ) have been shown to modulate AR transcriptional activity, often attenuating AR-driven proliferative signaling through effects on PI3K/Akt, epidermal growth factor receptor (EGFR), and related pathways [57,58,59]. In addition, AR signaling interfaces with multiple kinase-driven networks, including PI3K/Akt, protein kinase (MAPK), proto-oncogene tyrosine–protein kinase (Src), and Signal Transducer and Activator of Transcription 3 (STAT3) pathways, positioning AR as a central integrator rather than a linear oncogenic driver [46,56,57,58,59,60].

Mechanistically, AR signaling has been shown to interact with the PI3K/Akt pathway through reciprocal regulatory feedback, whereby AR action can modulate PI3K/Akt signaling, while activation of this pathway may in turn promote ligand-independent AR activation. Similarly, crosstalk with MAPK signaling can influence AR transcriptional activity by altering receptor phosphorylation and downstream gene expression programs. In addition, interactions between AR and STAT3 have been implicated in the regulation of genes involved in cell survival and immune-related pathways, further supporting the role of AR as an integrative node within complex oncogenic signaling networks [61,62,63]. These interactions are often mediated by post-translational modifications of AR, such as phosphorylation, which can influence receptor stability, nuclear localization, and transcriptional specificity, thereby fine-tuning AR-dependent gene expression programs.

Pharmacological inhibition of AR frequently results in compensatory activation of parallel signaling pathways, underscoring the adaptive nature of AR-associated networks in TNBC [46]. This suggests that AR signaling should not be considered as an isolated pathway but rather as part of a dynamic and interconnected signaling network, which may limit the efficacy of single-agent therapeutic strategies.

Clinically, AR expression in TNBC has been variably associated with prognosis, reflecting this biological heterogeneity. Associations with both favorable clinicopathological features and increased recurrence risk have been reported, further supporting the notion that AR signaling exerts divergent effects depending on the tumor context rather than acting as a consistent prognostic marker across all TNBC subtypes [64,65].

### 3.2. Androgen Receptor Splice Variants and Their Role in Treatment Resistance

In addition to full-length AR signaling, the presence of AR splice variants has been proposed as a potential mechanism contributing to resistance to AR-targeted therapies in TNBC [66]. Among these variants, variant 7 of the androgen receptor (AR-V7) has been the most extensively studied. AR-V7 lacks the ligand-binding domain but retains constitutive transcriptional activity independent of androgen stimulation [67].

Expression of AR-V7 has been associated with resistance to anti-androgen therapies in other hormone-driven malignancies and is increasingly being explored in the context of TNBC [68]. Mechanistically, AR-V7 has been shown to exhibit enhanced chromatin binding and preferential activation of genes involved in cell survival and proliferation, thereby sustaining AR-driven signaling despite pharmacological inhibition of the full-length receptor [69].

Recent experimental studies suggest that regulation of AR-V7 may occur independently of canonical AR expression. Caceres and colleagues (2025) reported persistent AR-V7 expression following AR silencing, suggesting the existence of distinct regulatory mechanisms governing splice variant expression [46]. Notably, although AR-V7 is considered ligand-independent, its expression was modulated by the hormonal environment, as exposure to DHT or E2 reduced AR-V7 levels in AR-negative TNBC cells. Anti-AR treatments similarly decreased AR-V7 expression without achieving complete suppression [63].

While these findings support a potential role for AR splice variants in mediating resistance to AR-targeted therapies, their clinical relevance in TNBC remains incompletely defined. Compared with prostate cancer, where AR-V7 has been extensively validated as a biomarker of resistance to anti-androgen therapies, its role in TNBC remains far less well established. Current evidence in TNBC is limited and largely derived from preclinical models or small patient cohorts, and its clinical utility as a predictive or prognostic biomarker has not yet been clearly demonstrated. These limitations highlight the need for further investigation to determine whether AR splice variants have a meaningful role in TNBC biology and therapeutic resistance and whether they may represent reliable biomarkers of resistance or actionable therapeutic targets in this breast cancer subtype [67].

## 4. Emerging Therapeutic Strategies for Triple-Negative Breast Cancer

Systemic chemotherapy remains a cornerstone of treatment for TNBC, often combined with surgery and, in selected cases, targeted therapies or immunotherapy. Although neoadjuvant and adjuvant chemotherapy regimens have improved outcomes in selected patients, pathological complete response (pCR) rates remain limited, and resistance to treatment continues to represent a major clinical challenge [70,71,72,73]. These limitations have prompted increasing interest in the identification of alternative therapeutic targets and biomarker-driven strategies.

Accumulating biological and translational evidence indicates that TNBC cannot be considered entirely hormone-independent. The presence of AR expression in a subset of tumors, together with preserved intracrine steroid metabolism, has provided the rationale for exploring androgen- and AR-targeted therapies in this disease [3,33]. However, the translation of this biological rationale into consistent clinical benefit has proven challenging.

Clinical trials evaluating AR antagonists and inhibitors of androgen biosynthesis in AR-positive TNBC have generally demonstrated modest activity, with limited progression-free survival (PFS) and heterogeneous clinical benefit rates [73,74]. This variability in clinical response likely reflects the absence of robust biomarkers of AR pathway activation and the underlying molecular heterogeneity of TNBC. While these agents are typically well tolerated and exhibit biological activity, their efficacy as monotherapy has been insufficient to support broad clinical application [75]. This pattern suggests that AR expression alone may be insufficient to define therapeutic vulnerability and that functional dependency on AR signaling likely varies across TNBC subtypes.

Combination strategies incorporating AR-targeted agents with chemotherapy, PI3K inhibitors, CDK4/6 inhibitors, or immunotherapy are currently being explored in early-phase trials. Although some studies have reported incremental improvements in clinical benefit, results remain preliminary and have not yet translated into durable responses or clear survival advantages [76,77,78,79,80,81,82,83,84,85,86,87,88]. An overview of clinical trials targeting androgen signaling in TNBC completed between 2019 and 2025 is summarized in Table 1. These findings further support the notion that effective therapeutic strategies will require targeting AR signaling within its broader network context rather than as an isolated pathway.

Clinical experience accumulated from studies evaluating AR antagonists, particularly enzalutamide, as well as inhibitors of androgen biosynthesis such as abiraterone acetate, orteronel, dutasteride, and seviteronel, confirms that these strategies are generally well tolerated but provide limited and short-lived clinical benefit when used as monotherapy in TNBC [89,90,91,92,93,94,95,96].

Consequently, increasing attention has shifted toward rational combination strategies aimed at overcoming resistance mechanisms and exploiting pathway crosstalk, including combinations with PI3K inhibitors, CDK4/6 inhibitors, chemotherapy, or immunotherapy [97,98,99,100].

Collectively, current clinical evidence suggests that AR-targeted therapies should be considered investigational approaches in TNBC. Future progress will depend on refined patient selection, improved characterization of AR signaling activity beyond immunohistochemical expression, and rational combination strategies informed by tumor molecular context.

The limited efficacy observed across clinical trials likely reflects several factors, including the biological heterogeneity of TNBC, the lack of robust biomarkers to identify AR-dependent tumors, and the context-dependent nature of AR signaling. In particular, reliance on AR expression alone by immunohistochemistry may not adequately capture functional pathway activation. In addition, compensatory signaling through pathways such as PI3K/Akt may attenuate the therapeutic impact of AR inhibition. Future strategies should therefore focus on improved patient stratification, the integration of functional biomarkers, and the development of rational combination therapies targeting key signaling interactions [73,74,75].

**Figure 3:**
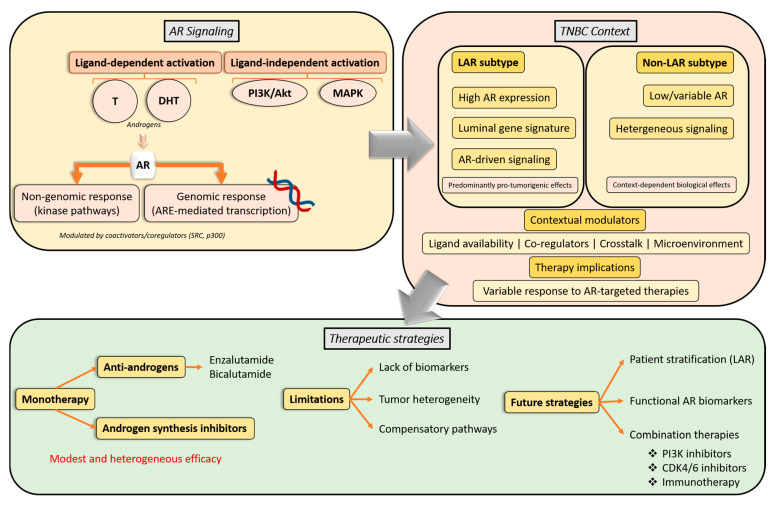
Overview of androgen receptor (AR) signaling, its context-dependent role in triple-negative breast cancer (TNBC), and therapeutic implications. AR activation occurs through ligand-dependent (testosterone, dihydrotestosterone) and ligand-independent pathways involving kinase signaling such as PI3K/Akt and MAPK. AR regulates both genomic and non-genomic responses, modulated by coactivators and coregulators. In TNBC, AR signaling shows subtype-specific roles, being more prominent in the luminal androgen receptor (LAR) subtype and more variable in non-LAR tumors. These context-dependent effects contribute to the limited and heterogeneous efficacy of AR-targeted therapies and support the development of improved patient stratification and combination strategies.

A schematic overview integrating AR signaling, its context-dependent role in TNBC subtypes, and the implications for AR-targeted therapeutic strategies is presented in Figure 3.

Taken together, these observations indicate that the clinical impact of AR-targeted therapies will depend on the identification of truly AR-dependent tumors and on strategies that account for pathway crosstalk and adaptive resistance mechanisms.

## 5. Innovations and Limitations in Breast Cancer Therapy: Insights from Animal Models

Experimental animal models are essential tools for investigating tumor biology and AR-related mechanisms in breast cancer, including TNBC [101,102,103]. This subtype, characterized by marked heterogeneity, aggressive behavior, and limited targeted treatment options, particularly benefits from the use of diverse preclinical models [1].

**Figure 4:**
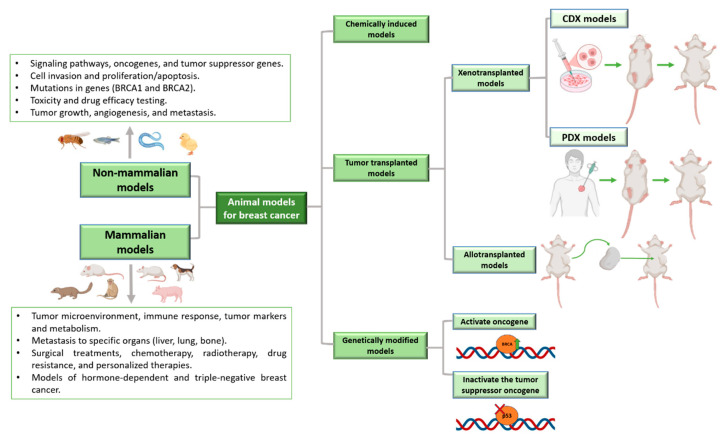
Overview of animal models used in breast cancer research. Schematic representation of commonly used models, including chemically induced tumors, cell-derived xenografts, and patient-derived xenografts (PDXs). Figure based on [100].

Murine models remain the most widely used platforms due to their experimental versatility and suitability for genetic manipulation and xenograft studies, including cell line-derived (CDX), patient-derived (PDX), and humanized mouse models [104,105] (Figure 4). These systems allow for the evaluation of tumor growth dynamics, therapeutic efficacy, and, in selected settings, tumor–immune system interactions.

Spontaneous mammary tumors observed in several species offer complementary advantages, including intact immune systems and naturally evolving tumor microenvironments [101]. Among these, canine mammary tumors have been explored as comparative models for TNBC due to certain shared histopathological and molecular features [106,107,108,109]. However, their ability to fully recapitulate human AR-positive TNBC remains limited. Accordingly, human-derived preclinical models that retain AR expression, such as cell line-derived and patient-derived xenografts, are more suitable for investigating AR-driven mechanisms and therapeutic responses [38,110,111].

In addition, established canine mammary tumor cell lines, such as IPC-366, derived from an inflammatory triple-negative mammary carcinoma, have been shown to express AR and share strong similarities with the human TNBC cell line SUM149, including tumor histology, hormone secretion profiles, metastatic behavior, and treatment response patterns [112]. These features make IPC-366 a relevant model for studying AR-driven mechanisms and therapeutic strategies in TNBC [35,38,113]. While informative, these findings should be interpreted with caution when extrapolating to human TNBC.

As therapeutic strategies for TNBC increasingly incorporate hormone-related pathways, including anti-androgen approaches, the use of preclinical models that retain AR expression and recapitulate steroid hormone signaling is essential [33,34]. Recent studies have shown that androgen-targeted therapies, such as dutasteride or bicalutamide combined with chemotherapy, can reduce tumor progression in both human and canine TNBC models [40,114].

Given the complexity of TNBC, the integration of complementary AR-characterized models across different stages of preclinical research remains critical for the development of effective therapeutic strategies [101].

## 6. Conclusions and Future Perspectives

This review summarizes current knowledge on the biological and clinical relevance of AR signaling in TNBC.

This subtype remains a heterogeneous disease with substantial therapeutic challenges, high recurrence rates, and poor prognosis. Growing experimental and translational evidence indicates that TNBC cannot be considered entirely hormone-independent, as local steroid hormone production and alternative hormone receptor signaling, including AR-mediated pathways, may contribute to tumor behavior. However, clinical evidence indicates that anti-androgen therapies in TNBC have demonstrated modest and heterogeneous activity, with limited progression-free survival benefits when used as monotherapy, underscoring the complexity of AR signaling and its context-dependent role in this disease. Accordingly, AR-targeted strategies are unlikely to be effective as monotherapy in unselected TNBC patients [114]. This limitation reflects not only the biological heterogeneity of TNBC but also the fact that AR expression does not necessarily equate to functional pathway dependency.

Future progress will depend on improved patient stratification, standardized assessment of AR activity beyond simple immunohistochemical expression, and the rational design of combination therapies that account for pathway crosstalk, tumor heterogeneity, and the hormonal microenvironment. In this regard, integration of molecular profiling, functional biomarkers, and preclinical models that faithfully recapitulate hormone signaling, such as murine and comparative canine models, will be essential to guide clinical translation.

In conclusion, while androgen and AR signaling represent biologically relevant components of TNBC, their therapeutic exploitation remains challenging. Ultimately, defining the contexts in which TNBC tumors are truly AR-dependent will be critical to determine whether targeting the androgen pathway can translate into meaningful clinical benefit for selected patient populations.

## Figures and Tables

**Table 1:** Clinical trials targeting androgens and the androgen receptor (2019–2025).

Clinical Trials of Androgen and Androgen Receptor Since 2019 to 2025					
Trial (NCT)	Phase	Population	Intervention	Key Outcomes	Ref
**NCT01889238**	II	Locally advanced or metastatic AR+ TNBC (≥10% AR)	Enzalutamide	CBR wk 16: 33.3%/24.6%. CBR wk 24: 28.2%/20.3%. PFS: 14.3/12.6 mo	[76]
**NCT02457910**	Ib/II	Stage IV AR+ TNBC	Taselisib + Enzalutamide	CBR: 0.357% vs. 0%. PFS: 3.4 mo	[77]
**NCT02689427**	IIb	AR+ TNBC	Enzalutamide + Paclitaxel	RCB-I: 25%; RCB-II: 33.3%; RCB-III: 25%.	[78]
**NCT03207529**	I	AR+ TNBC AR+ BC	Alpelisib + Enzalutamide	No results reported.	[79]
**NCT02368691**	II	Advanced AR+ TNBC	Enobosarm	CBR at wk 16: 5.6%. PFS: 1.9 mo DoR: 1.9 mo.	[80]
**NCT02971761**	II	Metastatic AR+ TNBC	Enobosarm + Pembrolizumab	pCR: 6.25%. PR: 6.25%. CBR wk 16: 25%	[81]
**NCT01990209**	II	Metastatic AR+ TNBC AR+ BC	Orteronel	ORR: 4.8%/0%. DCR: 4.8%/8.7%. PFS: 2 mo/1.8 mo OS: 10.2 mo/7.6 mo	[82]
**NCT00468715**	II	Metastatic ER-/PR-/AR+ BC	Bicalutamide	CBR: 19%. PFS: 12 wk	[83]
**NCT03055312**	III	Metastatic AR+ TNBC	Bicalutamide	No results reported.	[84]
**NCT02605486**	I/II	HR-negative metastatic BC	Palbociclib + Bicalutamide	Ongoing.	[85]
**NCT03090165**	II	Advanced AR+ TNBC	Ribociclib + Bicalutamide	No results reported.	[86]
**NCT06365788**	II	Metastatic AR+ TNBC	Abemaciclib + Bicalutamide	Recruiting.	[87]
**NCT02580448**	I/II	TNBC/HR+ BC	Seviteronel	No results reported.	[88]

Abbreviations: AR, androgen receptor; TNBC, triple-negative breast cancer; BC, breast cancer; HR+, hormone receptor-positive; CBR, clinical benefit rate; PFS, progression-free survival; OS, overall survival; ORR, objective response rate; DCR, disease control rate; RCB, residual cancer burden; pCR, pathological complete response; PR, partial response; DoR, duration of response.

## Data Availability

Data are contained within the article.

## Abbreviations

The following abbreviations are used in this manuscript:

A4	Androstenedione
AR	Androgen receptor
AR+ TNBC	Androgen receptor-positive triple-negative breast cancer
AR-V7	Variant 7 of androgen receptor
AREs	Androgen response elements
CDX	Cell line-derived xenograft
DBD	DNA-binding domain
DHEA	Dihydroepiandrostenedione
DHEA-S	Dihydroepiandrostenedione Sulfate
DHT	Dihydrotestosterone
E1SO4	Estrone sulphate
EGFR	Epidermal growth factor receptor
ERα	Estrogen receptor α
ERβ	Estrogen receptor β
GPER-1	G protein-coupled estrogen receptor 1
HER2	Human epidermal growth factor receptor 2
LAR	Luminal androgen receptor
LBD	Ligand-binding domain
MAPK	Protein kinase
NTD	N-terminal domain
pCR	Pathological complete response
PDX	Patient-derived xenograft
PFS	Progression-free survival
PR	Progesterone receptor
STAT3	Signal transducer and activator of transcription 3
Src	Proto-oncogene tyrosine–protein kinase
STS	Steroid sulfatase
TNBC	Triple-negative breast cancer
